# Caspase-3 Activation Correlates With the Initial Mitochondrial Membrane Depolarization in Neonatal Cerebellar Granule Neurons

**DOI:** 10.3389/fcell.2020.00544

**Published:** 2020-07-02

**Authors:** Edaena Benítez-Rangel, Mauricio Olguín-Albuerne, María Cristina López-Méndez, Guadalupe Domínguez-Macouzet, Agustín Guerrero-Hernández, Julio Morán

**Affiliations:** ^1^Departamento de Bioquímica, CINVESTAV-IPN, Mexico City, Mexico; ^2^División de Neurociencias, Instituto de Fisiología Celular, Universidad Nacional Autónoma de México, Mexico City, Mexico

**Keywords:** neuronal death, cerebellar granule neurons, calcium, endoplasmic reticulum, mitochondria, endoplasmic reticulum stress

## Abstract

In this study we evaluated the effect of the reduction in the endoplasmic reticulum calcium concentration ([Ca^2+^]_ER_), changes in the cytoplasmic calcium concentration ([Ca^2+^]_i_), alteration of the mitochondrial membrane potential, and the ER stress in the activation of caspase-3 in neonatal cerebellar granule cells (CGN). The cells were loaded with Fura-2 to detect changes in the [Ca^2+^]_i_ and with Mag-fluo-4 to measure variations in the [Ca^2+^]_ER_ or with TMRE to follow modifications in the mitochondrial membrane potential in response to five different inducers of CGN cell death. These inducers were staurosporine, thapsigargin, tunicamycin, nifedipine and plasma membrane repolarization by switching culture medium from 25 mM KCl (K25) to 5 mM KCl (K5). Additionally, different markers of ER stress were determined and all these parameters were correlated with the activation of caspase-3. The different inducers of cell death in CGN resulted in three different levels of activation of caspase-3. The highest caspase-3 activity occurred in response to K5. At the same time, staurosporine, nifedipine, and tunicamycin elicited an intermediate activation of caspase-3. Importantly, thapsigargin did not activate caspase-3 at any time. Both K5 and nifedipine rapidly decreased the [Ca^2+^]_i_, but only K5 immediately reduced the [Ca^2+^]_ER_ and the mitochondrial membrane potential. Staurosporine and tunicamycin increased the [Ca^2+^]_i_ and they decreased both the [Ca^2+^]_ER_ and mitochondrial membrane potential, but at a much lower rate than K5. Thapsigargin strongly increased the [Ca^2+^]_i_, but it took 10 min to observe any decrease in the mitochondrial membrane potential. Three cell death inducers -K5, staurosporine, and thapsigargin- elicited ER stress, but they took 30 min to have any effect. Thapsigargin, as expected, displayed the highest efficacy activating PERK. Moreover, a specific PERK inhibitor did not have any impact on cell death triggered by these cell death inducers. Our data suggest that voltage-gated Ca^2+^ channels, that are not dihydropyridine-sensitive, load the ER with Ca^2+^ and this Ca^2+^ flux plays a critical role in keeping the mitochondrial membrane potential polarized. A rapid decrease in the [Ca^2+^]_ER_ resulted in rapid mitochondrial membrane depolarization and strong activation of caspase-3 without the intervention of the ER stress in CGN.

## Introduction

The endoplasmic reticulum (ER) is one of the most important intracellular Ca^2+^ stores. It is well documented that a sustained reduction in the luminal [Ca^2+^] of the ER can result in a condition known as ER stress that eventually can lead to cell death (Boyce and Yuan, 2006; Szegedi et al., 2006; Sun et al., 2013). This condition may result in the activation of the ER stress receptor pathways. Particularly, IRE1 can lead to apoptotic cell death by the activation of JNK (Szegedi et al., 2006). We have previously shown that JNK is involved in the apoptotic cell death of cultured neonatal cerebellar granule neurons (CGN) induced by potassium deprivation (K5), but not by staurosporine (Ramiro-Cortés and Morán, 2009). The possible mechanisms relating ER stress activation with the apoptotic death remain to be elucidated. One possibility is that the cytoplasmic Ca^2+^ reduction induced by potassium deprivation could lead to a decrease of the luminal ER [Ca^2+^], which in turn could cause an ER stress in this model.

Alternatively, alterations in the transfer of Ca^2+^ from the ER to the mitochondria could be the reason behind CGN apoptotic death-induced by the decreased [Ca^2+^]_i._ It has been calculated that about half of the Ca^2+^ released from the ER is taken up by the mitochondria (Pacher et al., 2000; Lam and Galione, 2013) and that Ca^2+^ movement from the ER to the mitochondria via IP_3_R regulates energy production by increasing respiration and accelerating the Krebs cycle (Cardenas et al., 2010, 2015). It is then feasible that a reduction of Ca^2+^ movement from the ER to the mitochondria might result in cell death (Rizzuto et al., 2012).

Programmed cell death is critical for the physiology of the nervous system. Alterations in this program result in different neurological ailments; for instance, an excess of neuronal cell death leads to neurodegenerative diseases such as Parkinson’s, Alzheimer’s, among others. During development, a large proportion of CGN die (Wood et al., 1993) with features of apoptotic death (Lossi et al., 1998). CGN have been used as a model to study apoptosis during neuronal development. In that regard, it is known that calcium ions (Ca^2+^) are involved in the apoptotic death of CGN. These cells need a sustained Ca^2+^ influx for survival, which can be achieved by culturing cells in high K^+^ (25 mM, K25), which in turn activates voltage-gated Ca^2+^ channels (VGCC) resulting in a continuous Ca^2+^ influx and increased intracellular calcium concentration ([Ca^2+^]_i_) (Gallo et al., 1987; Moran and Patel, 1989; Alavez et al., 1996).

In support of the role played by sustained calcium influx in avoiding apoptosis in CGN, it has been shown that apoptotic cell death of CGN chronically cultured in K25 can be induced by blocking their VGCC (Kingsbury and Balazs, 1987; Balazs et al., 1988; Pearson et al., 1992; Copani et al., 1995). Thus, CGN prepared from postnatal rats maintained in the K25 medium can survive and mature in culture conditions. Moreover, CGN that had been chronically cultured in K25 would die by apoptosis when they are transferred to a non-depolarizing medium containing 5 mM KCl (K5) (Moran and Patel, 1989; D’Mello et al., 1993, 1997; Alavez et al., 1996; Ishitani et al., 1997; Nardi et al., 1997; Villalba et al., 1997; Morán et al., 1999).

The repolarization of plasma membrane potential by transferring cells to 5 mM K^+^ leads to the deactivation of VGCC. This, in turn, results in the inhibition of Ca^2+^ influx and subsequent cell death. A result that can be prevented by increasing the [Ca^2+^]_i_ with the addition of a Ca^2+^ ionophore (Morán et al., 1999). We have also found that caspase-3 transcription and activation induced by K5 is mediated by the fall in the [Ca^2+^]_i_ in CGN (Morán et al., 1999). In contrast to K5, the apoptotic death of CGN evoked by other conditions such as the incubation with staurosporine, a generalized kinase inhibitor, turned out to be Ca^2+^-independent. Despite the experimental evidence showing that a reduction in Ca^2+^ influx leads to apoptotic death for CGN, there is not enough information to propose a mechanism of action for Ca^2+^, particularly during the early phase of the apoptotic death in this model.

To test these possibilities, we have evaluated the effect of different inducers of apoptosis on Ca^2+^ release from the ER in cultured neonatal CGN and we have looked into the correlation between this calcium release and caspase-3 activation, mitochondrial depolarization, and ER stress. Based on the present results, we propose that the survival of CGN requires a sustained Ca^2+^ transfer from the ER to the mitochondria to keep this organelle polarized and the inhibition of this Ca^2+^ transfer leads to a rapid mitochondrial partial depolarization that in turn leads to apoptotic death in this neuronal survival model.

## Materials and Methods

### Cerebellar Granule Neurons Cultures

All animals used in this study were treated following the accepted standards of animal care and with the procedures approved by the local Committee of Research and Ethics of the Instituto de Fisiología Celular, Universidad Nacional Autónoma de México. The protocol used followed the Guidelines for the Care and Use of Mammals in Neuroscience as well as guidelines released by the Mexican Institutes of Health Research and the National Institutes of Health Guide for the care and use of laboratory animals (NIH Publication No. 8023, revised 1978). All efforts were made to minimize animal suffering and to reduce the number of animals used.

CGN cultures were prepared as previously described (Morán et al., 1999). Briefly, cell suspensions dissociated from 8 day-old rat cerebellum were plated at a density of 1.5 × 10^6^ or 2 × 10^6^ cells/ml in 12 or 6 well culture plates with or without coverslips coated with poly-L-lysine (5 μg/ml). They were maintained for 6–8 days *in vitro*. Culture medium contained basal Eagle’s medium supplemented with 10% (v/v) heat-inactivated fetal calf serum, 2 mM glutamine, 25 mM KCl, 50 U/ml penicillin, and 50 mg/ml streptomycin. This medium is denoted in the text as K25 and is the control condition. The culture dishes were incubated at 37 °C in a humidified 5% CO_2_/95% air atmosphere. Cytosine arabinoside (10 mM) was added 24 h after seeding to avoid the presence of non-neuronal cells. To trigger cell death, CGN were treated with staurosporine (0.5 μM), thapsigargin (2 μM), tunicamycin (20 μg/ml), and nifedipine (10 μM) or they were transferred to a medium identical to K25 except that KCl was 5 mM (it is denoted as K5).

### Simultaneous Recording of Changes in the Cytosolic and the ER Ca^2+^ Concentrations

CGN (6–7 days) seeded on coverslips (1.5 × 10^6^ or 2 × 10^6^ cells/ml) were incubated with MagFluo4-AM (0.25 μM) in the dark and at room temperature for 1 h. Subsequently, Fura2-AM (1.5 μM) was added for 1 h, without removing the first dye. Cells were washed with K25 for 10–30 min and the coverslips were mounted in a device into the cuvette. The dyes were excited at 340, 360, and 380 nm wavelength for Fura-2 and 495 nm wavelength for MagFluo-4 (Dagnino-Acosta and Guerrero-Hernández, 2009) and fluorescence emission was collected at 530 nm wavelength by using a QM-8 spectrofluorometer (PTI). Changes in both the [Ca^2+^]_i_ (fura-2) and [Ca^2+^]_ER_ (Magfluo-4) in response to the addition of staurosporine, thapsigargin, nifedipine, or tunicamycin were carried out in CGN maintained in K25. Ca^2+^ release from the ER for CGN in K25 was stimulated with either histamine or glutamate. Changes in the [Ca^2+^]_i_ and the [Ca^2+^]_ER_ were induced by exchanging K25 with K5 or vice versa.

To convert Fura-2 fluorescence ratios into [Ca^2+^]_i_, digitonin was added to obtain Rmax followed by EGTA (5 mM) to get Rmin. The background fluorescence was determined at the end by adding Mn^2+^, and this value was subtracted before doing the ratio 340/380. To correct for coverslip movements and the autofluorescence of staurosporine, the 360 nm fluorescence signal was subtracted from the 340 and 380 nm signals, as previously indicated (Benitez-Rangel et al., 2015). Briefly,

Ec (1): (λ_exc_ 340 - λ_exc_ 360) + C = λ_exc_ 340 corrected
Ec (2): (λ_exc_ 380 - λ_exc_ 360) + C = λ_exc_ 380 corrected

where C corresponds to 360 nm basal fluorescence.

These corrected values became intracellular Ca^2+^ using the following equation (3) (Grynkiewicz et al., 1985):

Ec (3): Ca^2+^ = K_d_ β [(R - R_min_)/(R_max_ – R)]

MagFluo-4 fluorescence was normalized using the initial signal as Fo. Since we do not know the resting level of the [Ca^2+^]_ER_ in CGN, then changes in the [Ca^2+^]_ER_ are indicated as ΔF/Fo.

### Detection of Mitochondrial Membrane Potential

Initially, we attempted to study changes in the mitochondrial [Ca^2+^] and for this reason, CGN were incubated with 1 μM rhod-2/AM for 1 h in the dark and at room temperature. However, this dye, in agreement with previous reports (Fonteriz et al., 2010), modified mitochondrial morphology when compared to the one observed with TMRE (Supplementary Figure S1). For this reason, we discarded the use of rhod-2 and decided to follow changes in the mitochondrial membrane potential with TMRE as a better indicator of the metabolic state of this organelle.

To this end, CGN grown for 6–7 days on coverslips, were incubated with TMRE (50 nM) in the dark and at room temperature for 30 min. The cells were washed with K25 for 5–10 min and the coverslip was placed in a cuvette using a homemade holder. The dye was excited at 549 nm and emission was recorded at 575 nm using a QM-8 spectrofluorometer (PTI). Initial fluorescence (Fo) was recorded, and then changes in mitochondrial membrane potential were induced by switching solutions (K25 to K5) and by adding different inducers of apoptosis in K25. At the end of the experiment, FCCP (20 μM) was added as a depolarization control. Negative values of ΔF/Fo reflect depolarization of mitochondrial membrane potential, as shown by the application of FCCP. Simultaneous changes in the [Ca^2+^]_i_ and the [Ca^2+^]_ER_ were obtained in the same CGN. Changes in the mitochondrial membrane potential with TMRE were acquired from the same cultured CGN, but using a different coverslip.

### Determination of Caspase-3 Activity

CGN cultured for 6–8 days were incubated with ER stressors (thapsigargin, tunicamycin) or cell death inducers (K25 to K5, staurosporine) for 6 h. Cells were placed on ice, lysed with caspase-3 assay buffer (50 mM HEPES, 5 mM DTT, and Triton X-100 1%), and the cell suspension was stirred for 30 min. Cells were scraped with a rubber cell scraper, and the cell lysate was recovered and kept frozen until the caspase-3 activity assay was carried out. Caspase-3 activity was determined using a fluorescent assay determined by a QM-8 spectrofluorometer (PTI) according to the manufacturer specifications, but with slight modifications as previously described (Morán et al., 1999; Benitez-Rangel et al., 2011). Briefly, determinations were carried out in assay buffer (20 mM HEPES, 5 mM DTT, 2 mM EDTA, and 0.1% Triton X-100, pH 7.4). For each determinations, assay buffer contained Ac-DEVD-AMC (caspase-3 substrate) with or without 2 μM Ac-DEVD-CHO (caspase-3 inhibitor) and 50 μl of cell lysate. The reactions were recorded for 20–30 min after the addition of the caspase substrate (1 μM) and cell lysate (30–60 mg/ml). Caspase-3 activity was the difference in fluorescence between the caspase-3 inhibitor being present or not. Basal caspase-3 activity was obtained from CGN that were kept in K25 without any treatment. Signal obtained from control cells (K25) was taken as one fold response.

### Western Blot

Cells were washed twice in ice-cold PBS and homogenized in lysis buffer (25mM Trizma, 50mM NaCl, 2% Igepal, 0.2% SDS, and complete protease and phosphatases inhibitors, pH7.4). The protein concentration of cellular homogenates was determined by using the Bradford method. A total of 40μg of soluble protein per lane were subjected to 15% SDS-PAGE and transferred to polyvinylidene difluoride membranes. Membranes were blocked with fat-free milk (5% in Tris-buffered saline (TBS)/Tween 20 (TTBS) buffer [100mM Trizma, 150mM NaCl, and 0.1% Tween, pH7.5]) and incubated overnight at 4 °C with the following specific primary antibodies: 1:1000 rabbit anti-eIF2α, 1:1000 mouse anti-GRP78, and 1:2000 mouse anti-β-Tubulin. β-Tubulin was used as a loading control.

Bands were visualized using chemiluminescence according to the manufacturer’s recommendations and exposed to Kodak BioMax-LightFilm. The optical density (O.D.) of each band was divided by the corresponding O.D. for β-tubulin and this ratio was compared to the one obtained from CGN extracts maintained in K25 (this value represents onefold response).

### Imaging CGN in the Confocal Microscope

CGN grown for 6–7 days on coverslips were incubated with the combination of TMRE (50 nM, 1 h) and MagFluo-4 (250 nM, 2 h) or with Rhod-2 (1 μM, 1 h) in the dark and at room temperature. Cells were washed with K25 for 5–10 min. The coverslip was mounted to a recording chamber placed on the stage of the microscope. The fluorescence images were collected using a confocal microscope Carl Zeiss LSM 700 with a 63x oil immersion objective. The excitation wavelengths were 488 nm for Mag-fluo-4 and 555 nm for Rhod-2 and TMRE.

### Statistical Analysis

Statistical analysis was done by using SigmaPlot 12.1 software. Data are expressed as means ± SEM. Pairwise comparison within multiples groups was made by analysis of variance (ANOVA) followed by the Holm Sidak *post hoc* test or by the non-parametric analysis Kruskal–Wallis followed by the Dunnett *post hoc* test; *p*-values less than 0.05 were considered statistically significant.

## Results

**FIGURE 1:**
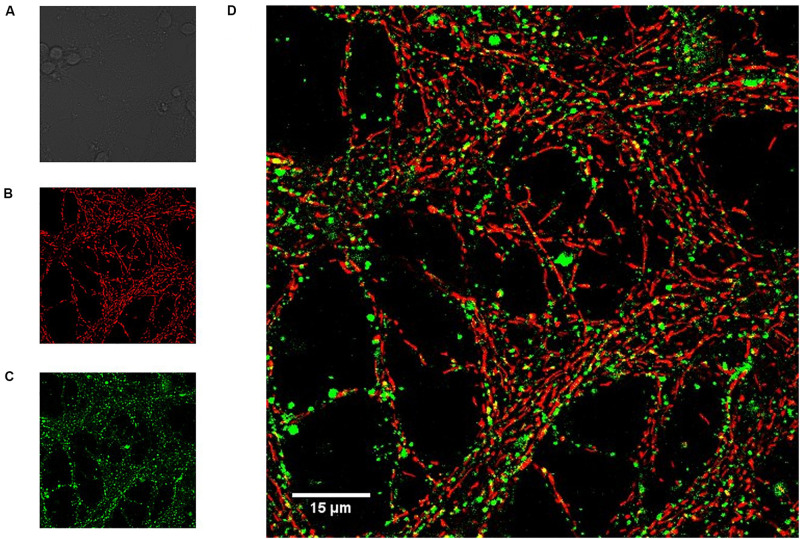
Magfluo-4 signal did not colocalize with mitochondria stained with TMRE in CGN. Cerebellar granule neurons (CGN) loaded with calcium indicator Magfluo-4 and mitochondrial membrane potential indicator TMRE were visualized with a Zeiss LSM 710 confocal microscope (600x). MagFluo-4 and TMRE dyes were located in different areas within the same CGN group. **(A)** Bright field, showing the soma of different CGN; **(B)** TMRE signal (red, mitochondria); **(C)** MagFluo-4 signal (green, high calcium areas); **(D)** the merged images showed a minimal colocalization of the labels as noted by the absence of yellow pixels.

Neonatal CGN were cultured on coverslips and loaded with both Mag-fluo-4/AM to determine luminal ER calcium concentration and with TMRE to measure the mitochondrial membrane potential and imaged with a confocal microscope (Figure 1). Cell bodies are seen in the bright field image (Figure 1A), but not in fluorescence images because they are out of focus. Neurites were heavily stained with both TMRE (Figure 1B) and Mag-fluo-4 (Figure 1C), the latter indicating regions of high luminal [Ca^2+^] that did not colocalize with the mitochondrial membrane potential indicator (Figure 1D). These data confirm that Mag-fluo-4 is not reporting mitochondrial deposits under our dye loading conditions and most likely is reporting changes in the luminal ER [Ca^2+^], so the Mag-fluo-4 signal is referred as the [Ca^2+^]_ER_. The simultaneous recording of CGN [Ca^2+^]_i_ and [Ca^2+^]_ER_ was obtained by loading cells with fura-2/AM and Mag-fluo-4/AM, respectively, and using a spectrofluorometer.

CGN that were in K5 displayed a normal resting [Ca^2+^]_i_ below 100 nM (Supplementary Figure S2), which is in line with the idea that these cells are repolarized (high membrane potential). Plasma membrane depolarization by adding K25 (Supplementary Figure S2A) produced an immediate increase in the [Ca^2+^]_i_ (blue trace). This elevation was followed by a slow rise in the [Ca^2+^]_ER_ (green trace) without any significant change in the mitochondrial membrane potential (red trace) for the initial 10 min after membrane depolarization. The application of thapsigargin, to inhibit the sarco/endoplasmic reticulum Ca^2+^-ATPase (SERCA) pump, resulted in a slow reduction in the [Ca^2+^]_ER_. In this case, the plasma membrane depolarization with K25 derived in a much larger increase in the [Ca^2+^]_i_ than the one observed in those CGN with active SERCA pump and as expected, no increase in the [Ca^2+^]_ER_ was attained (Supplementary Figure S2B). These data suggest that membrane depolarization of CGN activates voltage-gated calcium channels at the plasma membrane and a significant fraction of the Ca^2+^ entering the cell is sequestered in the ER by the action of SERCA pump. CGN in K25 were challenged with either histamine (400 μM) or glutamate (200 μM) to verify the ER Ca^2+^ store. Both agonists produced a similar initial increase in the [Ca^2+^]_i_. Nevertheless, the ER Ca^2+^ reduction was smaller and slower with histamine (Supplementary Figure S2C) than with glutamate (Supplementary Figure S2D). The addition of thapsigargin resulted in a significant decrease in the [Ca^2+^]_ER_ after the application of glutamate.

**FIGURE 2:**
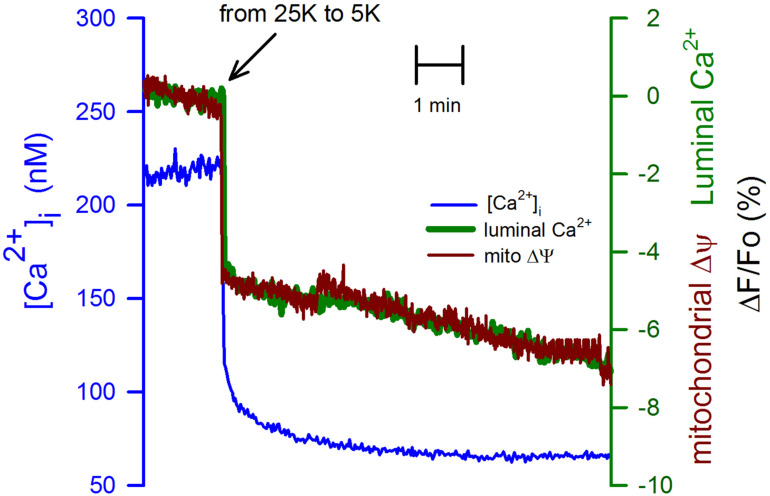
Switching from K25 to K5 decreased the [Ca^2+^]_i_, the [Ca^2+^]_ER_, and also associated with mitochondrial membrane depolarization. Simultaneous recordings of the [Ca^2+^]_i_ (blue trace) and the [Ca^2+^]_ER_ (green trace) are correlated in time with the mitochondrial membrane depolarization (red trace) induced by replacing K25 with K5. The synchronization of these records was carried out using the time when solutions were changed. The scale for the changes in the [Ca^2+^]_i_ is on the left-hand axis while the scale for the [Ca^2+^]_ER_ and mitochondrial membrane potential was the same (ΔF/Fo) and is shown on the right-hand axis. Average traces for Fura-2 ([Ca^2+^]_i_) and Magfluo-4 ([Ca^2+^]_ER_) from 11 independent experiments. Average mitochondrial membrane potential trace for 8 independent experiments.

CGN in K25 showed a significantly larger resting [Ca^2+^]_i_, above 200 nM, that was immediately decreased to below 100 nM by switching from K25 to K5 (Figure 2, blue trace). We verified that a mechanical stimulus by the change of solution could not be the explanation for the reduction in the [Ca^2+^]_i_ because switching solutions from K25 for another K25 did not have any apparent effect on the [Ca^2+^]_i_, the luminal [Ca^2+^]_ER_, or the mitochondrial membrane potential (Supplementary Figure S3). Interestingly, with practically the same time course, both the [Ca^2+^]_ER_ (Figure 2, green trace) and the mitochondrial membrane potential (Figure 2, red trace) were significantly decreased by replacing K25 with K5. Importantly, this effect of plasma membrane repolarization on mitochondria membrane potential was much faster but smaller than the decrease seen with FCCP. The latter produces a complete mitochondrial membrane depolarization (Supplementary Figure S4).

**FIGURE 3:**
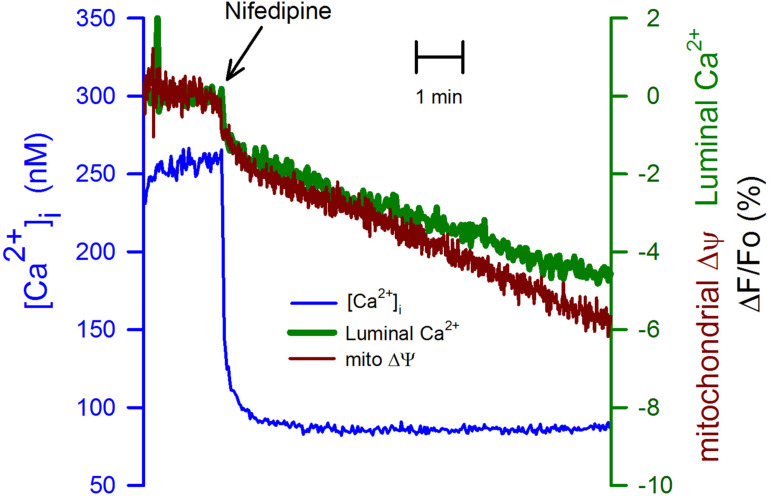
Inhibition of VGCCs with nifedipine while inhibiting Ca^2+^ entry to the cytoplasm did not have the same effect on the [Ca^2+^]_ER_ or the mitochondrial membrane potential. CGN in K25 were exposed to nifedipine (10 μM) at the indicated time (black arrow) to mimic the deactivation of VGCCs as produced by going from K25 to K5. The reduction in the [Ca^2+^]_i_ (blue trace) was of the same amplitude as the one seen with K5 (Figure 2). However, the [Ca^2+^]_ER_ (green trace) was resistant to the reduction in the [Ca^2+^]_i_ suggesting the presence of Ca^2+^ entry via a dihydropyridine-resistant VGCCs which is also replenishing the ER Ca^2+^ store. The mitochondrial membrane depolarization (red trace) showed the same time course of the decrease in the [Ca^2+^]_ER_. Average traces (*n* = 8) for the [Ca^2+^]_i_ and the [Ca^2+^]_ER_. Average TMRE trace for six independent experiments.

It is known that cell depolarization and the subsequent activation of voltage-gated calcium channels are responsible for Ca^2+^ entering the cytoplasm. We employed nifedipine (10 μM) to inhibit this Ca^2+^ entry. This dihydropyridine reduced the [Ca^2+^]_i_ similarly, as seen with K5 (compare Figure 3 with Figure 2). Nevertheless, the effect of nifedipine on the [Ca^2+^]_ER_ was smaller and slower when compared to K5 (Figure 3). Additionally, the mitochondrial membrane depolarization (Figure 3, red trace) followed the same time course as the one observed for the [Ca^2+^]_ER_. These data suggest that there are other voltage-gated calcium channels, different from the dihydropyridine receptors, and that they efficiently load the ER with Ca^2+^. Moreover, it appears that mitochondria membrane potential strongly depends on the Ca^2+^ loading of the ER. Our data suggest that plasma membrane VGCCs, different from dihydropyridine-sensitive L-type calcium channels, participate in Ca^2+^ entry to the cytoplasm and that these channels, but not those sensitive to nifedipine, are in close connection with the SERCA pump of the ER. These data also suggest that the mitochondrial membrane potential is affected by rapid reductions in the luminal [Ca^2+^]_ER_.

**FIGURE 4:**
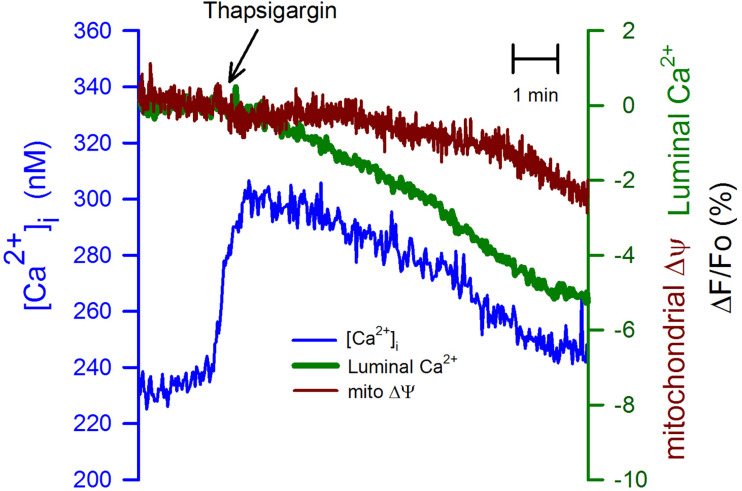
Thapsigargin uncoupled the depletion of the [Ca^2+^]_ER_ from the mitochondrial membrane depolarization. Thapsigargin (2 μM) was applied at the indicated time (black arrow) to CGN in K25. This inhibitor of the SERCA pump produces a transient elevation of the [Ca^2+^]_i_ (blue trace), but a much slower reduction in the [Ca^2+^]_ER_ (green trace) and even slower reduction of the mitochondrial membrane potential (red trace). Average recordings (*n* = 9) for the [Ca^2+^]_i_ and the [Ca^2+^]_ER_. Average trace for TMRE (*n* = 4).

We have used thapsigargin (2 μM) -a potent and specific inhibitor of SERCA pump (Lytton et al., 1991)- to test the connection between the ER and mitochondria in depolarized CGN. The inhibition of SERCA pumps produced an immediate but transient increase in the [Ca^2+^]_i_ (Figure 4, blue trace) with a delayed reduction in the luminal [Ca^2+^]_ER_ and even more delayed and a smaller reduction in the mitochondria membrane potential. These data suggest that mitochondrial membrane potential is sensitive to the luminal [Ca^2+^]_ER_, but an increase in the [Ca^2+^]_i_ could prevent mitochondrial depolarization.

**FIGURE 5:**
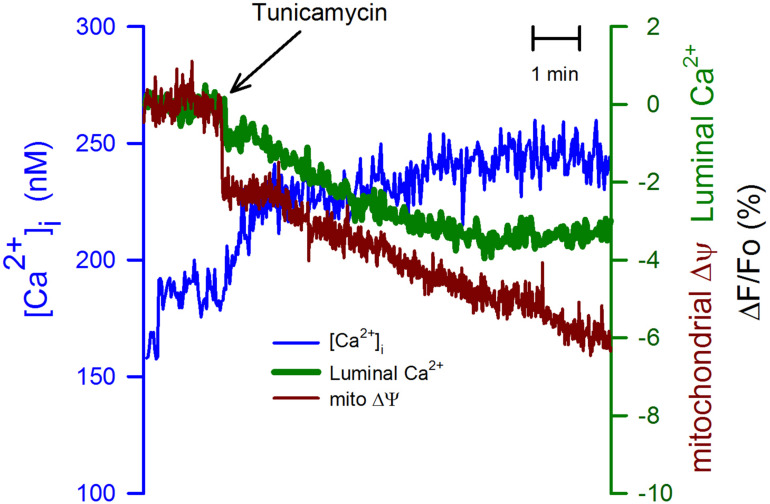
Tunicamycin had a stronger effect on the mitochondrial membrane potential than on the [Ca^2+^]_ER_. The ER stressor tunicamycin (20 μg/ml) was applied at the time indicated (black arrow) to CGN in K25. Tunicamycin increased the ([Ca^2+^]_i_) and it also produced a slow reduction in the [Ca2+]ER, but the mitochondrial membrane potential displayed a biphasic response, an initial fast decrease followed by a much more gradual decline in the mitochondrial membrane potential (red trace). In this case, tunicamycin displayed a stronger effect on the mitochondrial membrane potential than on the two different calcium concentrations. Average traces (*n* = 6) for all three various indicators.

To further study the connection between the ER and the mitochondria, we have used tunicamycin (20 μg/ml). This is an inhibitor of protein glycosylation, which also produces ER stress (Liu et al., 1997). Tunicamycin induced a slow but sustained elevation in the [Ca^2+^]_i_ that might result from the partial inhibition of the SERCA pump activity (Figure 5, blue trace). In this case, the mitochondrial membrane potential displayed a rapid reduction, followed by a slower rate of depolarization. Tunicamycin also produced an immediate and more gradual decrease in the [Ca^2+^]_ER_ (Figure 5, green trace). The observation that the time course between the mitochondrial membrane potential and the luminal [Ca^2+^]_ER_ is similar suggests that the luminal [Ca^2+^]_ER_ regulates mitochondria membrane potential. A situation also observed for K5 and nifedipine.

**FIGURE 6:**
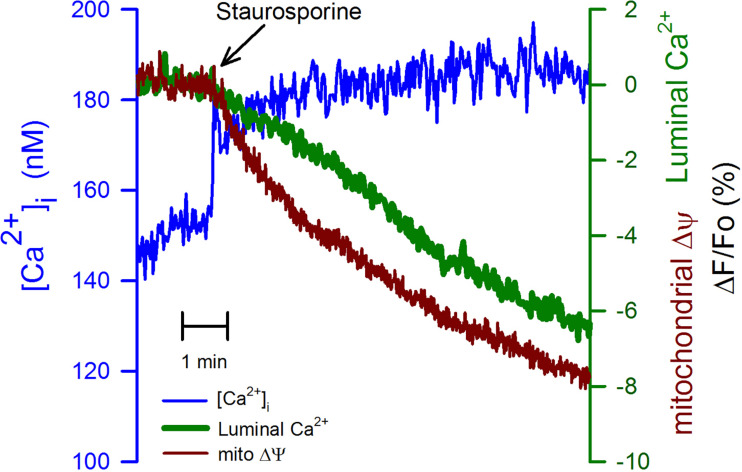
Staurosporine reduces mitochondrial membrane potential faster than the [Ca^2+^]_ER_. The application of staurosporine (1 μM), a strong apoptosis inducer, to CGN in K25 at the time indicated (black arrow) produced a fast increase in the [Ca^2+^]_i_ (blue trace) trailed by an evident depolarization of the mitochondrial membrane potential (red trace) trailed by a slower reduction in the [Ca^2+^]_ER_ (green trace). Average traces (*n* = 6) for all three different indicators.

We have studied the effect of staurosporine (1 μm), a nonspecific inhibitor of kinases, which also has a well-established role as an inducer of apoptosis (Tamaoki et al., 1986; Deshmukh and Johnson, 2000). This kinase inhibitor produced a sustained increase in the [Ca^2+^]_i_ (Figure 6, blue trace), the origin of this response was not further investigated. This response was followed by a slower but continuous reduction in the mitochondrial membrane potential (Figure 6, red trace) in association with a delayed decline in the luminal [Ca^2+^]_ER_. The reduction in the mitochondrial membrane potential triggered by staurosporine preceded the decrease in the [Ca^2+^]_ER_. Therefore, the reduction in the [Ca^2+^]_ER_ appears cannot fully explain the staurosporine-induced mitochondrial depolarization. The nature of the reduction in the [Ca^2+^]_ER_ induced by staurosporine was not further determined, however, it is clear that the time course for this reduction is quite different from the associated increase in the [Ca^2+^]_i_.

**FIGURE 7:**
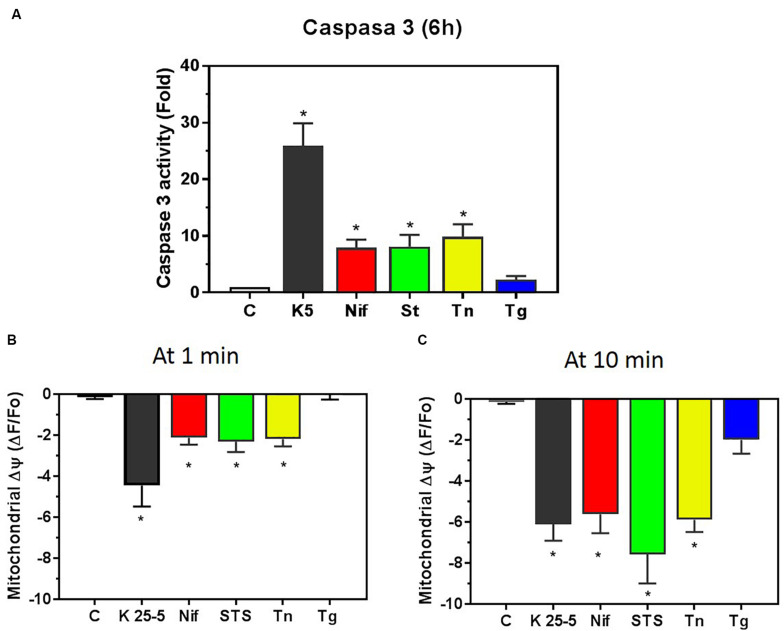
Caspase-3 activation by different inducers of apoptosis correlated with the early mitochondrial membrane depolarization. **(A)** Caspase-3 activity was determined in CGN lysates obtained after 6 h incubation with the following chemicals nifedipine (10 μM, red bar), staurosporine (1μM, green bar), tunicamycin (20 μg/ml, yellow bar) and thapsigargin (2 μM, blue bar). Additionally, CGN were incubated for 6 h with K5 (black bar), and caspase-3 activity was measured in cell lysates as indicated in section “Materials and Methods.” The caspase-3 activity for CGN in K25 was considered the basal level. Values indicate the average fold-increase normalized to the control ± SEM of 4 independent experiments. *p < 0.05 vs. control, C. **(B)** This panel shows the extent of mitochondrial membrane depolarization at 1 min after the addition of the different inducers of cell death. Bars show the same color code as in **(A)**. Values are means ± SEM of 4–6 independent experiments. *p < 0.05 vs. the level before the addition of the stimulus. **(C)** The mitochondrial membrane depolarization observed 10 min after the application of the indicated inducer of apoptosis. Note that thapsigargin-induced depolarization was not significant even at 10 min incubation time. Bars show the same color code as in **(A)**. Values indicate the means ± SEM of 4–11 independent experiments. *p < 0.05 vs. the level before the addition of the stimulus. Data shown in **(B,C)** come from the TMRE traces shown in Figures 2–6, and Supplementary Figure S5 compares the different time courses of mitochondria depolarization.

Both K5 and staurosporine induce apoptosis in these cells. We have studied the increase in caspase-3 activity caused by K5 in comparison with other inducers of cell death. CGN cultured in K25 were either switched to K5 for 6 h (Figure 7A, solid bar) or were incubated for 6 h with nifedipine (10 μM, Figure 7A red bar), staurosporine (1 μM, Figure 7A green bar), tunicamycin (20 μg/ml, Figure 7A yellow bar) or thapsigargin (2 μM, Figure 7A blue bar), and activity of caspase-3 was assessed as previously described (Morán et al., 1999; Benitez-Rangel et al., 2011). Incubation in K5 showed strong activation of caspase-3, while nifedipine produced a four-times smaller activation of this protease, similar to staurosporine and tunicamycin. Interestingly, thapsigargin did not significantly increase the activity of caspase-3. These data show that K5 is the most potent activator of caspase-3 in CGN, while the other conditions activate the protease to a lower extent, and thapsigargin is ineffective for the same incubation time.

Mitochondria is a critical element in the activation of caspase-3, thus, we decided to compare the effect of these chemicals on the mitochondrial membrane potential that had been previously recorded. The same pattern emerged as for activation of caspase-3 when compared with the initial (1 min) mitochondrial membrane depolarization induced by K5 (Figure 7B, solid bar), nifedipine (Figure 7B, red bar), staurosporine (Figure 7B, green bar), tunicamycin (Figure 7B, yellow bar), and thapsigargin (Figure 7B, blue bar). Remarkably, the mitochondria membrane depolarization is the same for all conditions at 10 min, except for thapsigargin (Figure 7C). Therefore it is only the initial mitochondrial membrane depolarization, and not the extent of this depolarization (Figure 7C), that better predicts the activation of caspase-3 by these five different inducers of apoptosis in CGN. These inducers of apoptosis displayed a different time course for the mitochondrial membrane depolarization (Supplementary Figure S5). K5 produced the fastest mitochondrial membrane depolarization, while staurosporine, tunicamycin, and nifedipine had a similar rate of reduction in the mitochondria membrane potential, and thapsigargin had the slowest time course. This time point of 1 min appears to correlate better for all five different conditions in the activation of caspase-3 because the mitochondrial membrane depolarization was the same at 10 min and yet the activation of caspase-3 was not the same 6 h later. Thapsigargin was unable to depolarize mitochondria at minute one, and accordingly, there was no activation of caspase-3 6 h later. These data suggest that it is the kinetics of mitochondrial membrane depolarization, and not the extent of depolarization, that predicts the extent of caspase-3 activation in CGN.

Interestingly, the 10 min level of mitochondria membrane depolarization (Figure 7C), the changes in the [Ca^2+^]_i_ (Supplementary Figure S6), and the extent of the reduction in the luminal [Ca^2+^]_ER_ (Supplementary Figure S7) did not show any correlation with the activation of caspase-3 in CGN. Collectively, these data suggest that membrane depolarization (K25) activates nifedipine-resistant VGCCs that are closely associated with the superficial ER, which in turn accumulates Ca^2+^ via SERCA pump and releases this ion to the mitochondria. It appears that this constant Ca^2+^ flux from the ER to the mitochondria is essential to keep mitochondria membrane potential and CGN survival. Accordingly, a drastic interruption of this Ca^2+^ flux would lead to the activation of caspase-3, which eventually results in cell demise.

**FIGURE 8:**
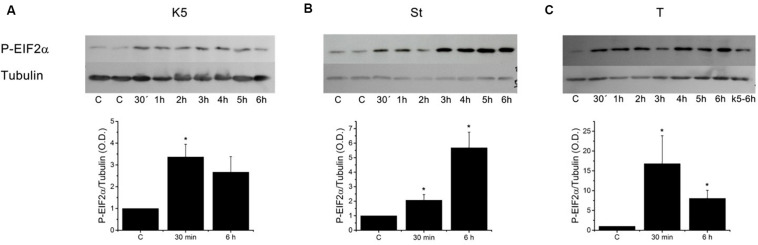
Different inducers of apoptosis triggered eIF2α phosphorylation in CGN. Cerebellar granule neurons were incubated with **(A)** K5 or treated with **(B)** staurosporine (0.5 μM, St) and **(C)** thapsigargin (2 μM, T) for times from 30 min to 6 h and the activation of PERK was determined based on the phosphorylation of eIF2α (∼38 kDa) and tubulin (∼52 kDa) was used as a loading control. Densitometric analysis was carried out for the control condition (CGN in K25) and 30 min and 6 h for each treatment. Thapsigargin was the most effective activator of PERK at the earliest time tested. Data are mean ± SEM. **(A)** (*p < 0.05, ANOVA, *n* = 4). **(B)** (*p < 0.05, Kruskal–Wallis, *n* = 4). **(C)** (*p < 0.05, Kruskal–Wallis, *n* = 3).

Since the decline in the [Ca^2+^]_ER_ produces ER stress that eventually could result in cell death (Tabas and Ron, 2011; Urra et al., 2013), we decided to study whether this reduction, more than mitochondrial membrane depolarization, could be the reason behind apoptosis in CGN. The cells were incubated in K5, or K25 with 0.5 μM staurosporine or 2 μM thapsigargin, and PERK activation was assessed by determining phosphorylation of eIF2α (Figure 8). All these three conditions significantly activated PERK at 30 min, which was the earliest time point evaluated. Thapsigargin was the most potent activator of PERK (Figure 8C), while staurosporine resulted in an intermediate effect, and K5 induced the smallest phosphorylation of eIF2α. These data corroborate that these three different conditions, that decreased the luminal [Ca^2+^]_ER_ also activated PERK kinase reflecting the activation of ER stress.

Importantly, the observed activation of PERK did not correlate with the activation of caspase-3 because thapsigargin, the most effective activator of PERK, barely activated caspase-3 at 6 h. Moreover, we have used GSK2606414, a potent inhibitor of PERK (Axten et al., 2012), to study the role of this kinase in the induction of apoptosis in CGN. GSK2606414 at 20 μM did not modify the expression levels of eIF2a (Supplementary Figure S8A) and yet fully inhibited its phosphorylation by either K5 (Supplementary Figure S8B) or thapsigargin (Supplementary Figure S8C). However, this PERK inhibitor did not improve CGN viability after 12, 24, and 48 h incubation with these three different inducers of apoptosis (Supplementary Figure S9). Moreover, these data corroborated that thapsigargin, a potent inducer of ER stress, is a weak inducer of cell demise. These data suggest that ER stress was not the reason behind the activation of caspase-3 and cell death in CGN.

It has been shown that ER stress produces adaptive responses that should allow cells to cope with the stress and avoid cell demise program. One of these responses is an increase in the expression of GRP78 or BiP. Nevertheless, none of the conditions that induce ER stress, based on the activation of PERK, led to an increased expression level of GRP78 (Supplementary Figure S10 ). This situation was the same, whether at 30 min or 6 h of incubation with K5, staurosporine, or thapsigargin. Unexpectedly, staurosporine led to an apparent reduction in the expression of GRP78 at 6 h (Supplementary Figure S10B). These data suggest that an increase in Bip expression cannot explain the lack of correlation between the induction of ER stress and activation of caspase-3 in CGN. Overall, these data discard ER stress as the cause for cell death in CGN and point to a rapid mitochondrial membrane depolarization due to a reduction in the Ca^2+^ release from the ER as the main reason for cell demise in this type of neurons.

## Discussion

We have studied the role played by Ca^2+^ in the induction of cell death in CGN by recording changes in the [Ca^2+^]_i_, the luminal [Ca^2+^]_ER_, and the mitochondrial membrane potential. We have found that the initial rate of mitochondrial membrane depolarization better predicts the extent of caspase-3 activation using five different cell death-inducing conditions in CGN. Nevertheless, all these five conditions, K5 (repolarization of plasma membrane potential), staurosporine, thapsigargin, nifedipine, and tunicamycin, have different mechanisms to induce cell death. Additionally, the ER stress does not seem to be associated with the activation of caspase-3 in CGN. Our data support the scenario where cell depolarization (25K), which is necessary for CGN survival, associates with sustained activation of VGCCs, increased [Ca^2+^]_i_, a higher level of the luminal [Ca^2+^]_ER_, and a larger mitochondrial membrane potential.

The use of nifedipine, to inhibit L-type VGCCs, has shown that this type of channel in CGN is fully responsible for the increase in the [Ca^2+^]_i_ in depolarizing conditions (K25). This is the case since both conditions, i.e., the addition of nifedipine in depolarizing medium (K25) or the repolarization of CGN by switching from K25 to K5, produced precisely the same reduction in the [Ca^2+^]_i_. These data argue that only L-type VGCCs are involved in the increase in the [Ca^2+^]_i_ induced by K25. Indeed, it has been shown that L-type VGCCs have a voltage activation curve more negative than the dihydropyridine-insensitive channels (Wheeler et al., 2012) in superior cervical ganglion neurons (SCGN).

Using Mag-fluo-4, we have corroborated that plasma membrane depolarization produced an increase in the luminal [Ca^2+^]_ER_ that required the activity of the SERCA pump while the plasma membrane repolarization resulted in a rapid reduction of the luminal [Ca^2+^]_ER_. However, the more interesting observation was a similar rate of decline for [Ca^2+^]_ER_ and [Ca^2+^]_i_, but only with K5 and not with nifedipine in K25. Nifedipine decreased the [Ca^2+^]_i_ very rapidly, but not the luminal [Ca^2+^]_ER_. These data argue for the presence of nifedipine-resistant VGCCs that are in the plasma membrane, but clustered just above the SERCA pump in the ER. This arrangement facilitates nifedipine-insensitive VGCCs to load the ER better than the L-type VGGCs (de Juan-Sanz et al., 2017). Indeed, the inhibition of the SERCA pump with thapsigargin produced a more significant increase in the [Ca^2+^]_i_ when CGN were depolarized by switching from K5 to K25, indicating that the ER is capturing a substantial amount of the Ca^2+^ entering into CGN via active VGCCs. It has been shown in SCGN that inhibition of the SERCA pump with thapsigargin produces a much larger [Ca^2+^]_i_ response when nimodipine-insensitive VGCCs were activated instead of the nimodipine-sensitive channels (Wheeler et al., 2012). These data were interpreted to indicate that the ER was positioned just beneath the plasma membrane where the dihydropyridine-insensitive channels (CaV2) were located and that Ca^2+^ entry mediated by these channels was loading ER Ca store by the action of SERCA pumps. Based on the data shown here, it appears then that the location of the SERCA pump underneath the dihydropyridine-insensitive VGCCs channels, observed for SCGN, is also present in CGN.

The functional interaction between VGCCs and ER and mitochondria seems to be highly complex. A recent study in cultured hippocampal neurons concluded that L-type VGCCs act in a bimodal way on the bioenergetics of the mitochondria depending on the magnitude of the stimulation of the VGCCs (Hotka et al., 2020). During low neuronal activity, VGCCs would promote an increase of [Ca^2+^]_ER_, which transfer Ca^2+^ to mitochondria provoking an increase in mitochondrial respiration. In contrast, high activity conditions lead to a marked direct increase of Ca^2+^ transport to mitochondria, without the participation of ER that affects mitochondrial respiration (Hotka et al., 2020). It has been shown that a high Ca^2+^ influx is associated with CGN survival. However, it is not clear whether this survival was the consequence of a high level in the ER or of Ca^2+^ uptake by mitochondria. The Ca^2+^ reduction in the ER results in triggering ER-stress response, and this, in turn, can trigger apoptosis. We have found that thapsigargin was the most effective way of provoking ER-stress, but the least effective activator of caspase-3. Moreover, the inhibitor of PERK did not improve survival for K5 condition. Together these data suggest that the reduction in the luminal [Ca^2+^]_ER_ due to the VGCC deactivation is not the explanation for the associated CGN cell death.

We used Rhod-2 to determine whether the repolarization induces a change of the levels of Ca^2+^ in the mitochondria. However, it has been described that this indicator deteriorates mitochondria, a situation that was observed in CGN. Another reliable way to assess the activity of mitochondria is to analyze the membrane potential; in this case, the repolarization of CGN resulted in an evident mitochondria depolarization, although of a much smaller magnitude than the one produced by a mitochondrial uncoupler. Interestingly, the rate of this membrane depolarization, and not the extent, showed a clear correlation with the amount of caspase-3 activated by the different inducers of cell death. For instance, K5 was the largest activator of caspase-3 and produced the fastest mitochondrial depolarization, while thapsigargin that activated the smallest amount of caspase-3 did not produce any early mitochondrial depolarization. Collectively, these data suggest that continuous Ca^2+^ supply by the ER to mitochondria is the explanation behind the prosurvival activity of K25 in CGN. It has been shown that Ca^2+^ transfer from the ER to mitochondria has a prosurvival effect in cancer cells and our results suggest that a similar situation occurs in CGN.

## Data Availability Statement

The datasets generated for this study are available on request to any of the corresponding authors.

## Ethics Statement

The animal study was reviewed and approved by the Animal Care and Use Committee of the Instituto de Fisiología Celular, Universidad Nacional Autónoma de México (protocol number JMA120-17).

## Author Contributions

EB-R carried out the experiments, contributed to analyze and interpret the data, participated in the design of the study, and to the draft of the manuscript. MO-A and GD-M carried out some of the experiments. ML-M contributed to obtaining and processing of images. AG-H and JM conceived the study, participated in its design, and contributed to draft the manuscript. All authors contributed to the article and approved the submitted version.

## Conflict of Interest

The authors declare that the research was conducted in the absence of any commercial or financial relationships that could be construed as a potential conflict of interest.
