# Supplementary material for: Caspase-3 Activation Correlates With the Initial Mitochondrial Membrane Depolarization in Neonatal Cerebellar Granule Neurons
Source: Front Cell Dev Biol. 2020 Jul 2;8:544. doi: 10.3389/fcell.2020.00544 (PMC7343937; doi:10.3389/fcell.2020.00544)
Supplement: Supplementary file 1 [file Data_Sheet_1.pdf]

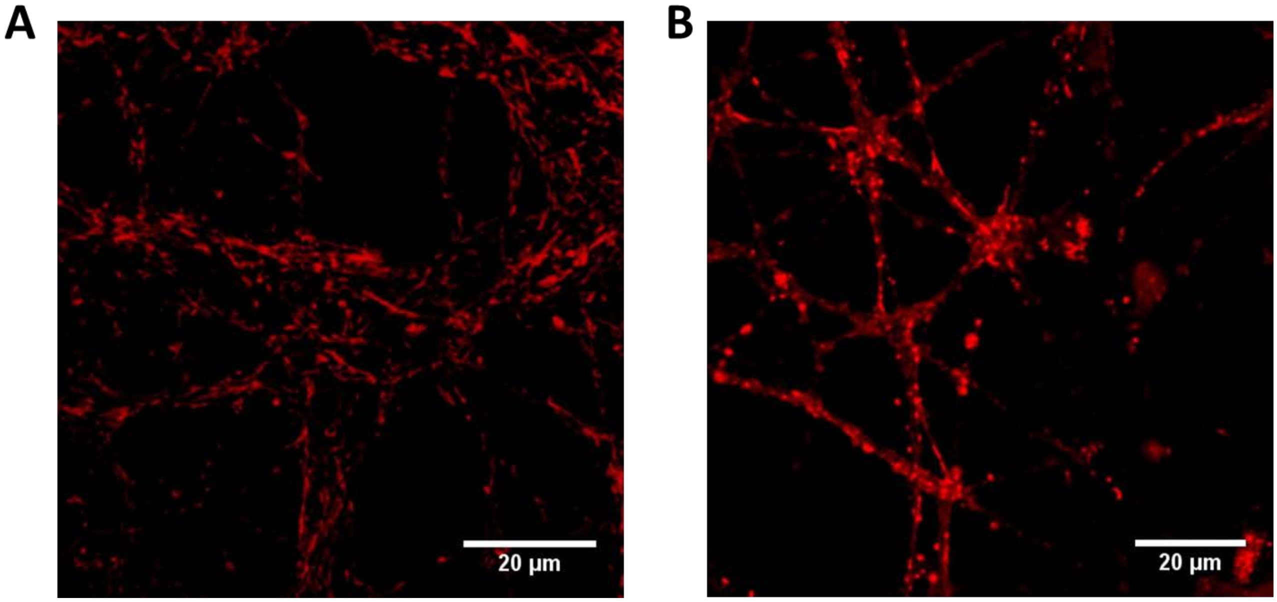

Figure S1. Rhod-2 modifies mitochondrial morphology, but not TMRE. CGN loaded with mitochondrial membrane potential indicator TMRE (A) or with the mitochondrial calcium indicator Rhod-2/AM (B) were visualized with a Zeiss LSM 710 confocal microscope. Both indicators use rhodamine as their fluorophore; nevertheless, Rhod-2 (B) resulted in rounded and fragmented mitochondria when compared with the images obtained using TMRE (A). These morphological alterations have been previously described in other cell types, and it appears to be due to the presence of the Ca^{2+} binding moiety in Rhod-2 since both indicators use Rhodamine. For this reason, we decided to use TMRE instead of Rhod-2 to indicate the metabolic state of the CGN mitochondria.

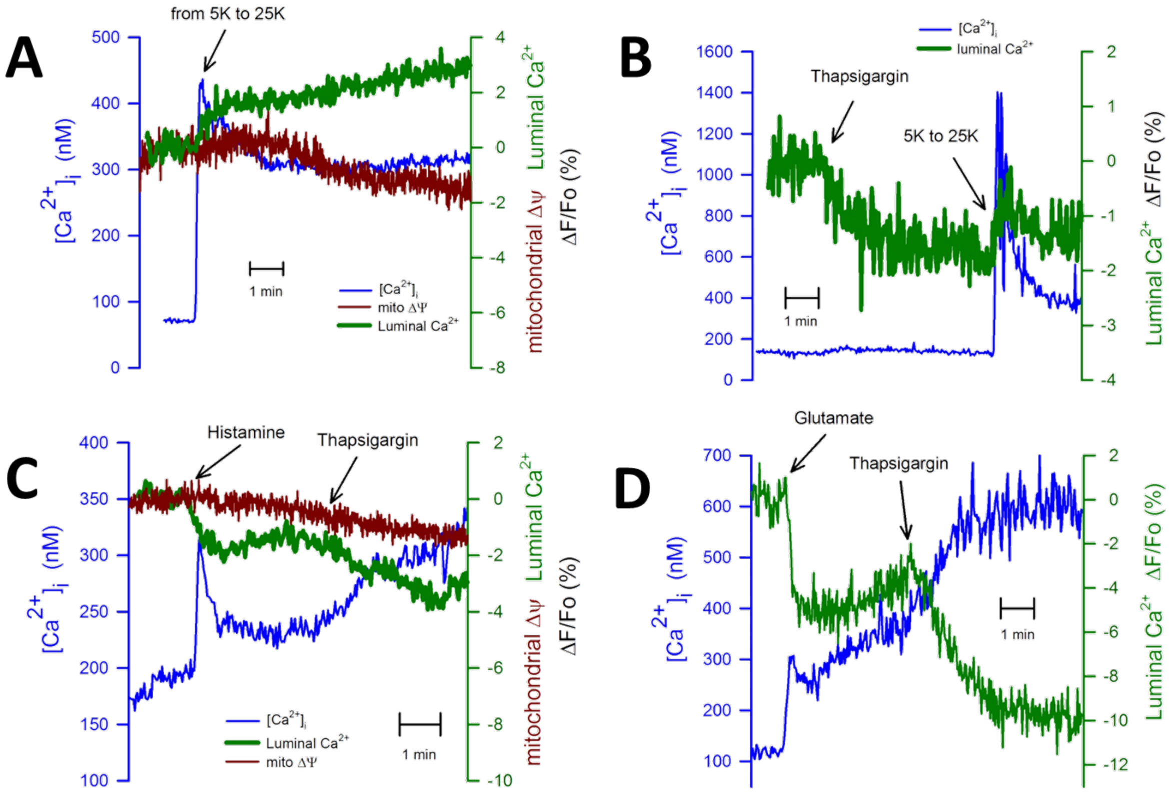

Figure S2. Changes in the $[Ca^{2+}]_i$ and $[Ca^{2+}]_{ER}$ induced by potassium depolarization, thapsigargin, histamine, or glutamate observed in CGNs cultured in K5. Simultaneous recordings of the $[Ca^{2+}]_i$ (Fura-2, blue trace), the $[Ca^{2+}]_{ER}$ (Magfluo-4, green trace), and mitochondrial membrane potential (A, C; TMRE, red trace). (A) CGNs were in K5, and the medium was switched to K25 (black arrow), resulting in a large increase of the $[Ca^{2+}]_i$, a noticeable elevation of the $[Ca^{2+}]_{ER}$ and a non-significant reduction in the mitochondrial membrane potential (n = 5). (B) CGNs cultured in K5 displayed a clear reduction in the $[Ca^{2+}]_{ER}$ due to the application of thapsigargin (2 µM, black arrow) with no increase in the $[Ca^{2+}]_i$. Once the reduction in the $[Ca^{2+}]_{ER}$ was stabilized, the K5 was replaced with K25 at the indicated time (black arrow). Depolarization of CGNs resulted in a much larger elevation of the $[Ca^{2+}]_i$ than when SERCA pumps were active (A). In the presence of thapsigargin, the $[Ca^{2+}]_{ER}$ did not increase in response to K25, as it was the case when SERCA pumps were functioning (A). The changes shown is a typical response from n = 19. The fact that membrane depolarization produces a much larger increase in the $[Ca^{2+}]_i$ when SERCA pumps are inhibited implies that the ER is buffering much of the Ca^{2+} entering CGNs via the activation of VGCCs. (C) The application of histamine (400 µM, black arrow) induced a small and transient decrease of the $[Ca^{2+}]_{ER}$ (green trace) with a transient increase in the $[Ca^{2+}]_i$ (blue trace). The subsequent application of thapsigargin (2 µM, black arrow) resulted in a further reduction of the $[Ca^{2+}]_{ER}$ and a sustained increase of the $[Ca^{2+}]_i$. Representative traces (n = 6). These changes in the calcium concentrations barely modified the mitochondria membrane potential (red trace). Representative recordings (n = 3). (D) The application of glutamate (200 µM, black arrow) resulted in a much larger decrease in the $[Ca^{2+}]_{ER}$ when compared to histamine but a similar initial increase in the $[Ca^{2+}]_i$. Glutamate produced a sustained elevation of the $[Ca^{2+}]_i$ that was further amplified by the application of thapsigargin (2 µM, black arrow) in combination with a further decline in the $[Ca^{2+}]_{ER}$. Representative traces (n = 3).

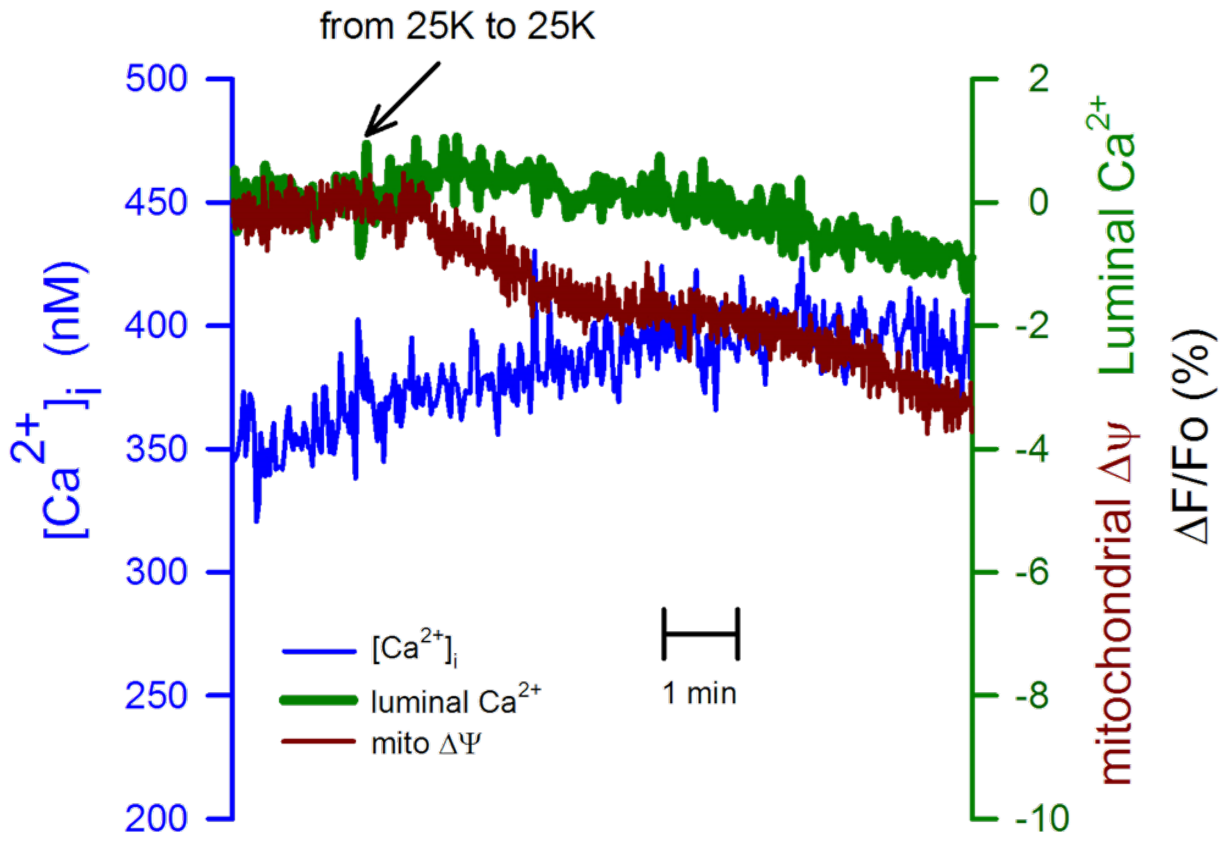

Figure S3. The mechanical stimulus due to solution changes is not responsible for the modification seen in the $[Ca^{2+}]_i$, the $[Ca^{2+}]_{ER}$, and the mitochondrial membrane potential. CGNs placed in K25 were recorded during switching solution for a different K25 to determine whether the mechanical stimuli due to the solution exchange was enough to trigger the changes observed in the $[Ca^{2+}]_i$, the $[Ca^{2+}]_{ER}$ and the mitochondrial membrane potential. The changes in the $[Ca^{2+}]_i$ and the $[Ca^{2+}]_{ER}$ were prolonged and not significant. This mechanical stimulus produced a small reduction of the mitochondrial membrane potential of similar magnitude to the one seen by the addition of thapsigargin (n = 4). We do not have an explanation for this small reduction.

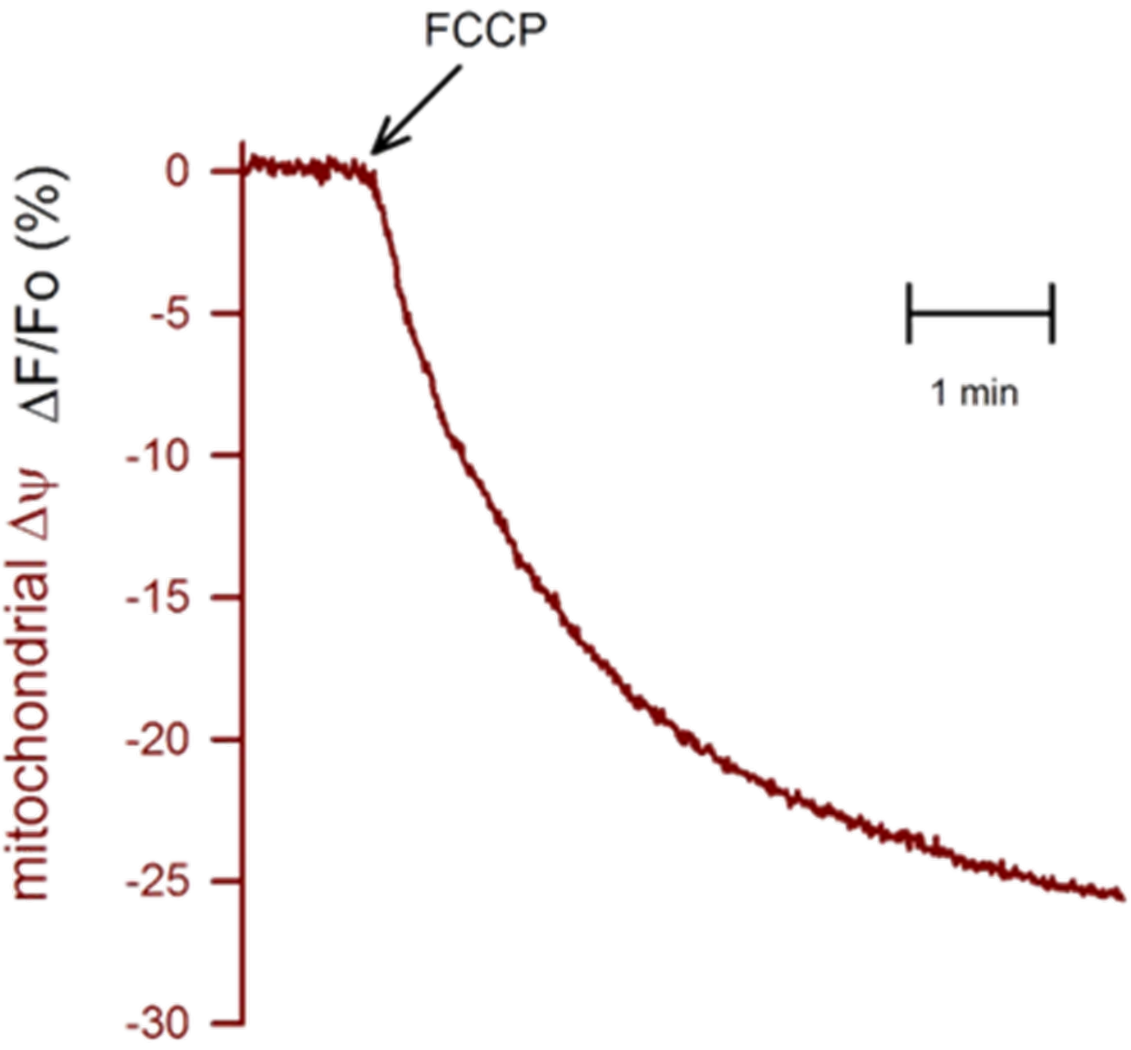

Figure S4. Time course of the mitochondrial membrane depolarization induced by the uncoupler FCCP in CGNs. CGNs in K25 were loaded with TMRE, and the uncoupler FCCP (20 μM) was added at the time indicated (black arrow). FCCP produced an immediate reduction in the mitochondrial membrane potential that was much larger but slower than the one observed by switching from K25 to K5. The trace shown is an average of n=17 experiments. The FCCP-induced reduction rate of TMRE fluorescence is slower than K5. However, K5-induced depolarization is faster than FCCP. This means that K5 depolarizes only a small fraction of mitochondria or a larger fraction with a smaller depolarization in CGNs.

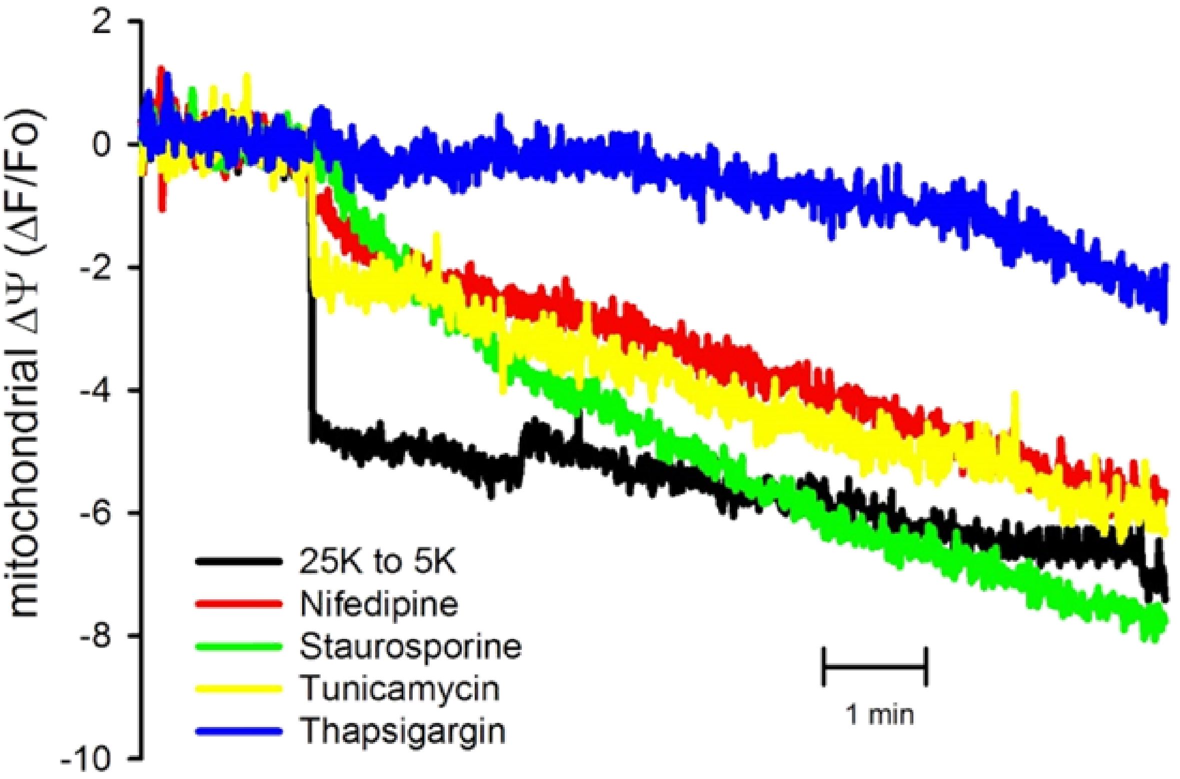

Figure S5. Comparison of mitochondrial membrane depolarization time courses for the five different cell death inducers (K25 to K5, nifedipine, staurosporine, tunicamycin, and thapsigargin). These traces are the average mitochondrial membrane potential recorded with TMRE and shown in figures 2 through 6. CGNs loaded with the mitochondrial membrane potential indicator TMRE were exposed to K5 (black trace), nifedipine (10 µM, red trace), staurosporine (1µM, green trace), tunicamycin (20 µg /ml, yellow trace) or thapsigargin (2 µM, blue trace), and changes in TMRE fluorescence were recorded for 10 min. Notice that the time courses were different among the five different inducers of cell death. However, mitochondrial depolarization at 10-min (6%) was similar for all inducers except thapsigargin, and this level is smaller than the one induced by FCCP (25%) that represents complete depolarization (Supplementary Figure S4).

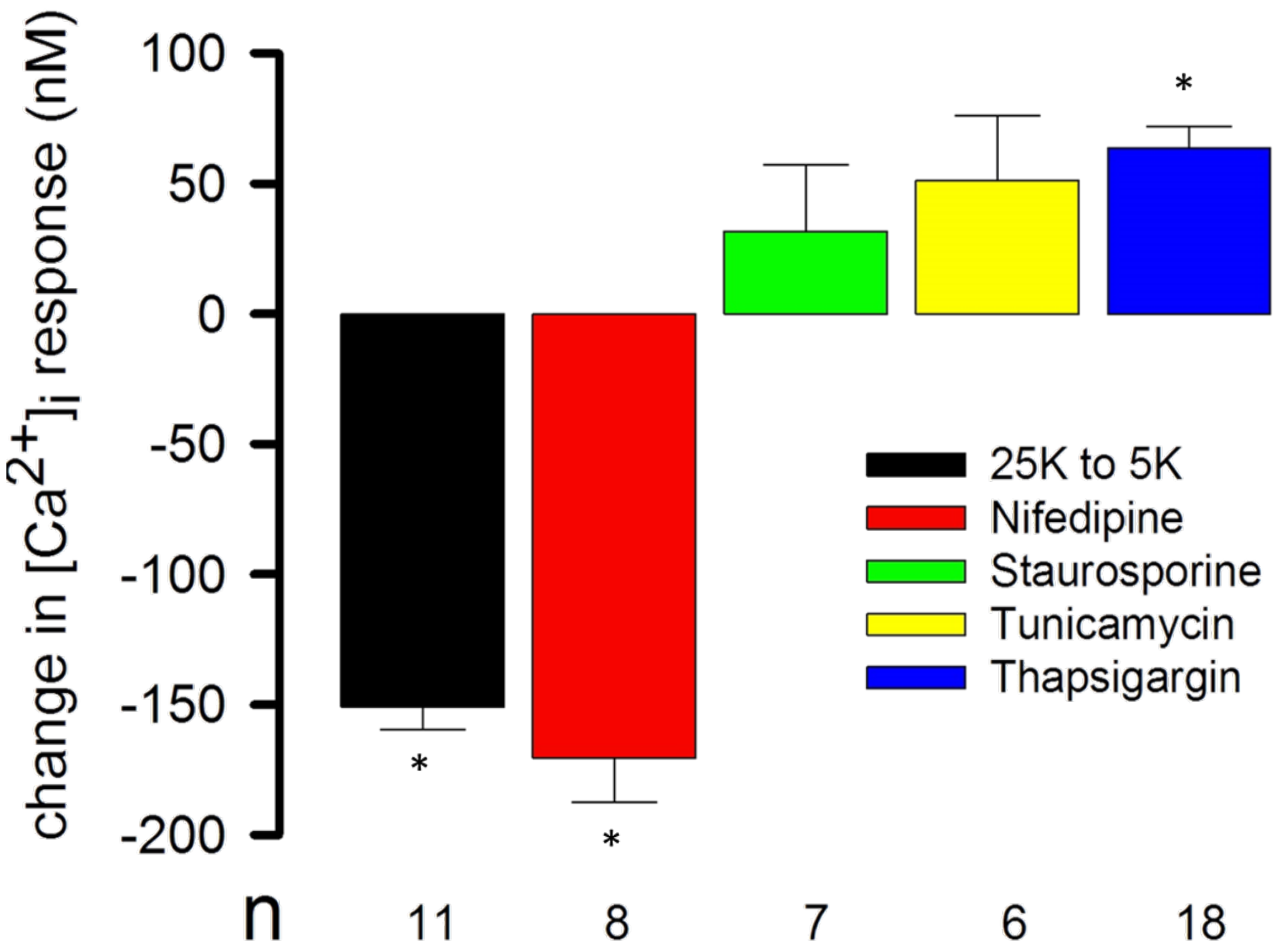

Figure S6. Disparate changes in the $[Ca^{2+}]_i$ in response to **K5, nifedipine, staurosporine, tunicamycin, and thapsigargin.** CGNs were challenged with the indicated five different conditions and the average change respect to the basal $[Ca^{2+}]_i$ is shown after 10 min. The conditions were K5 (black bar), nifedipine (10 µM, red bar), staurosporine (1µM, green bar), tunicamycin (20 µg /ml, yellow bar) and thapsigargin (2 µM, blue bar). Data show that these conditions produced three different kinds of responses on the $[Ca^{2+}]_i$, K5 and nifedipine decreased $[Ca^{2+}]_i$, staurosporine and tunicamycin did not have any substantial effect on the $[Ca^{2+}]_i$ and thapsigargin resulted in a significant increase of the $[Ca^{2+}]_i$. Bars show the mean ± SEM for the indicated (n) number of independent experiments. * $p<0.05$ vs. basal. These data suggest that the changes in the $[Ca^{2+}]_i$ do not correlate with the activation of caspase-3 four hours later by the same inducers.

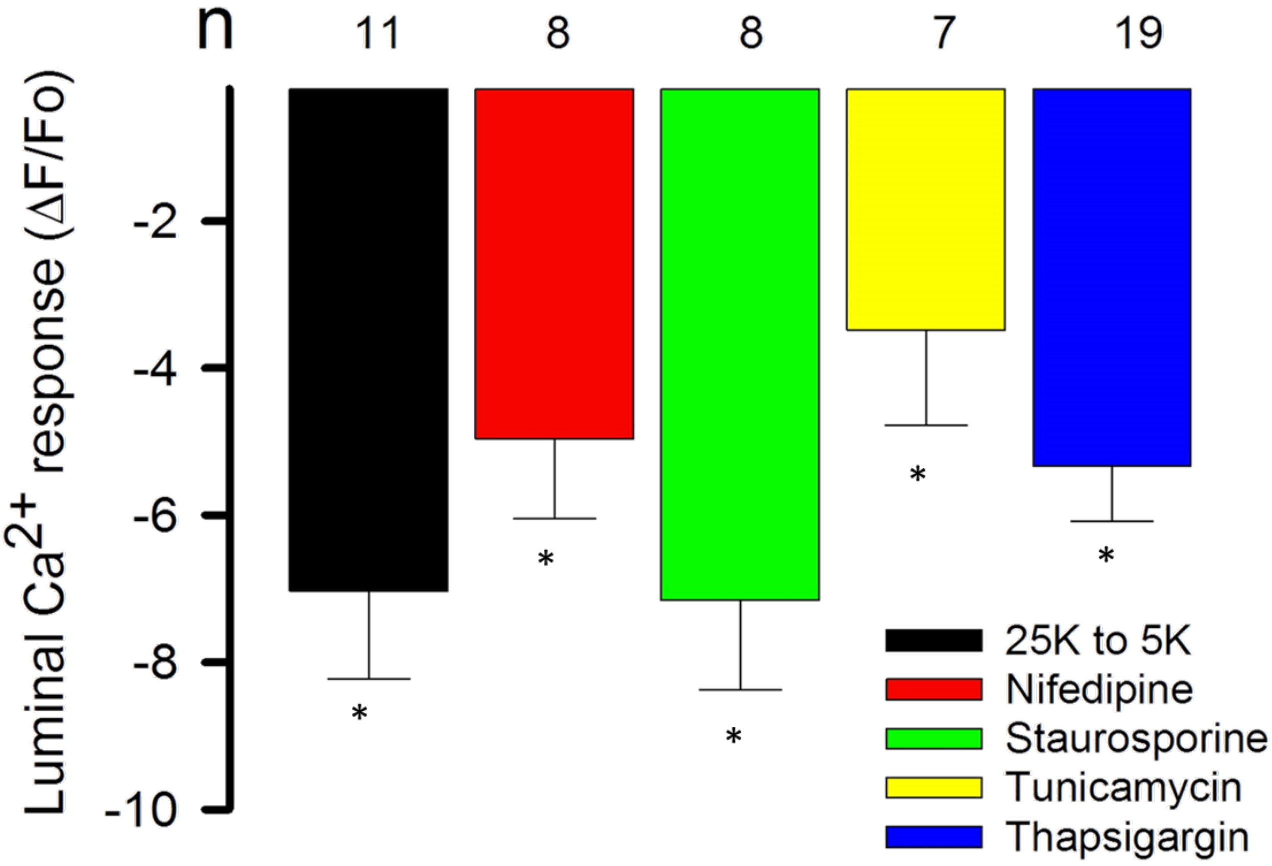

Figure S7. All inducers of apoptosis decreased the $[Ca^{2+}]_{ER}$**.** The $[Ca^{2+}]_{ER}$ attained after 10 min incubation period. CGNs had been exposed to K5 (black bar), nifedipine (10 µM, red bar), staurosporine (1µM, green bar), tunicamycin (20 µg /ml, yellow bar) and thapsigargin (2 µM, blue bar) and the average reduction in the $[Ca^{2+}]_{ER}$ are plotted as the mean ±_SEM for the indicated n number of independent experiments. * $p<0.05$ vs. basal. All different conditions significantly decreased the $[Ca^{2+}]_{ER}$ in an uncorrelated manner to the changes in the $[Ca^{2+}]_i$ or mitochondrial membrane potential.

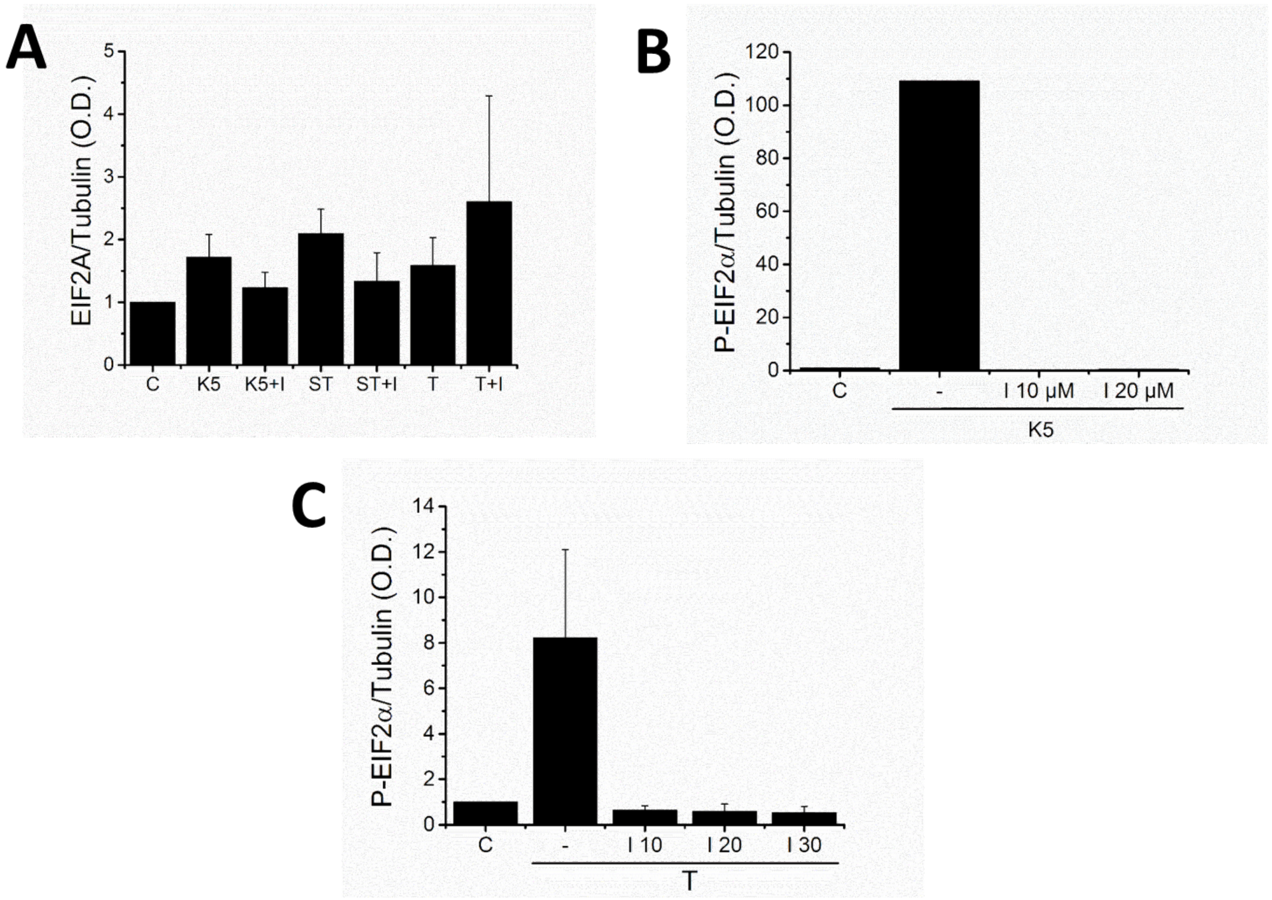

Figure S8. Effect of PERK inhibition on eIF2α phosphorylation induced by K5, staurosporine, and thapsigargin. CGNs cultured in K25 were exposed to K5, staurosporine (1μM, ST), or thapsigargin (2 μM, T) for 6 h and changes in the total amount eIF2α and its phosphorylation level were evaluated by Western blot as indicated in Methods. The control level was considered the one obtained for CGNs in K25 (C). Panel A shows the effect of a PERK inhibitor, GSK2606414 (10 μM, I) on changes in the total amount of eIF2α induced by K5, staurosporine, and thapsigargin. (B) The presence of either 10 or 20 μM GSK2606414 (I) of the PERK inhibitor fully reversed the eIF2α phosphorylation induced by K5. (C) The inhibitor of PERK, GSK2606414 (I) from 10 to 30 μM fully inhibited the eIF2α phosphorylation induced by thapsigargin. Bars show the densitometric ratio between eIF2α and tubulin that were normalized to the control ratio. Data are the mean ± SEM of 4 independent experiments.* $p<0.05$ vs. control.

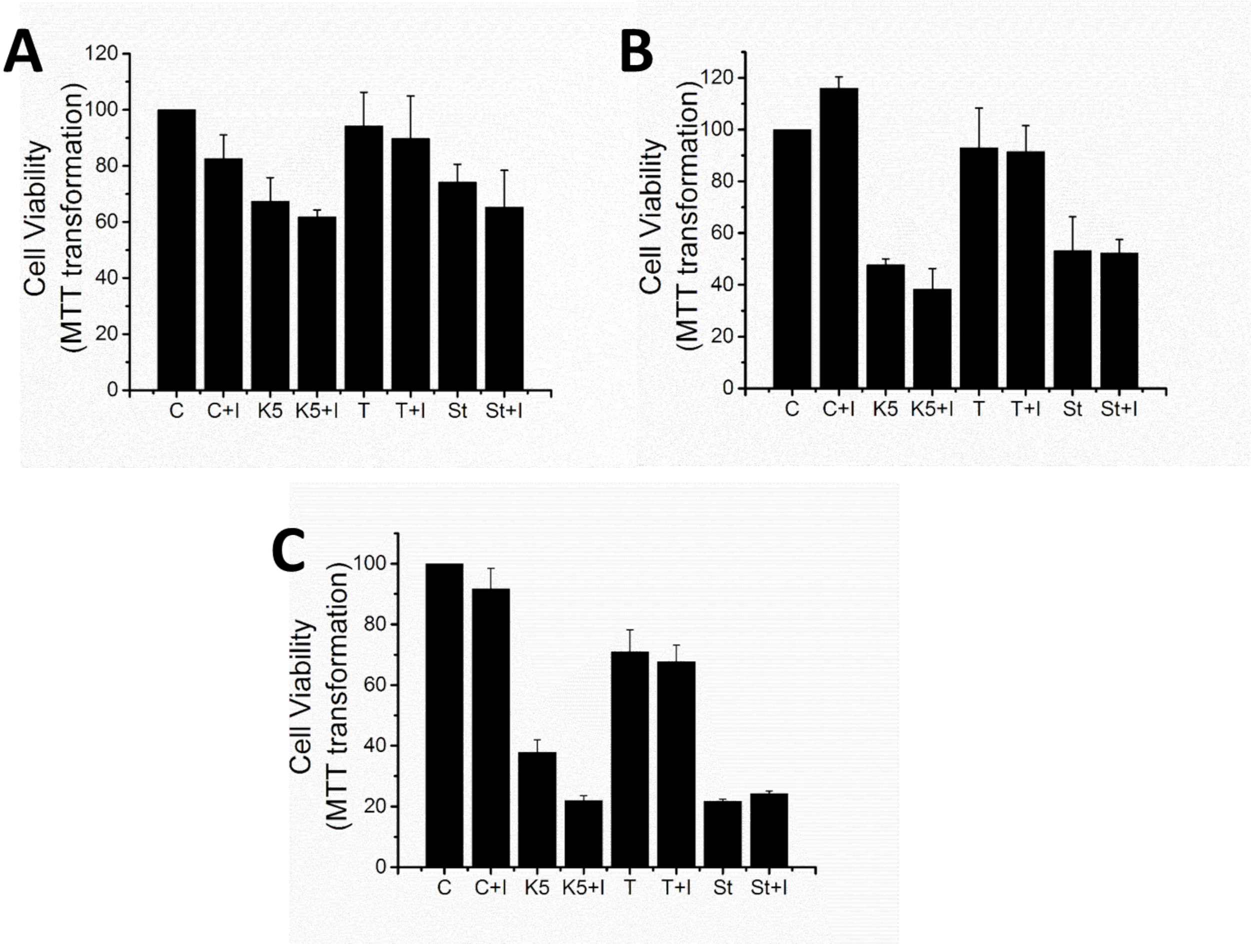

Figure S9. PERK inhibition with GSK2606414 did not reverse the decrease in GCN survival caused by K5, staurosporine, and thapsigargin. CGNs in K25 were exposed to K5, thapsigargin (2 μM, T), or staurosporine (1μM, St) in the absence or presence of 30 μM GSK2606414 (I). Cell viability was evaluated as indicated below for incubation periods of 12 (A), 24 (B), and 48 h (C). Control (C). Data are mean ± SEM of 4 independent experiments. Both K5 and staurosporine decreased CGN viability at 24 and 48 hours while this was not the case for thapsigargin. The PERK inhibitor did not improve CGN viability in any of the conditions tested. Cell viability was determined by the conversion of MTT (3-[4,5-dimethylthiazol-2-yl]-2,5 diphenyl tetrazolium bromide) into formazan crystals. MTT (0.25 mg/ml) was added to CGNs for 10 min at 37 ^{o}C in 5% CO_2-95% air atmosphere; the formazan blue produced from MTT was extracted with 100% DMSO and quantified spectroscopically at 560 nm excitation wavelength. Cell viability is expressed as the percent of MTT transformation of cells cultured in K25 (100%, C)

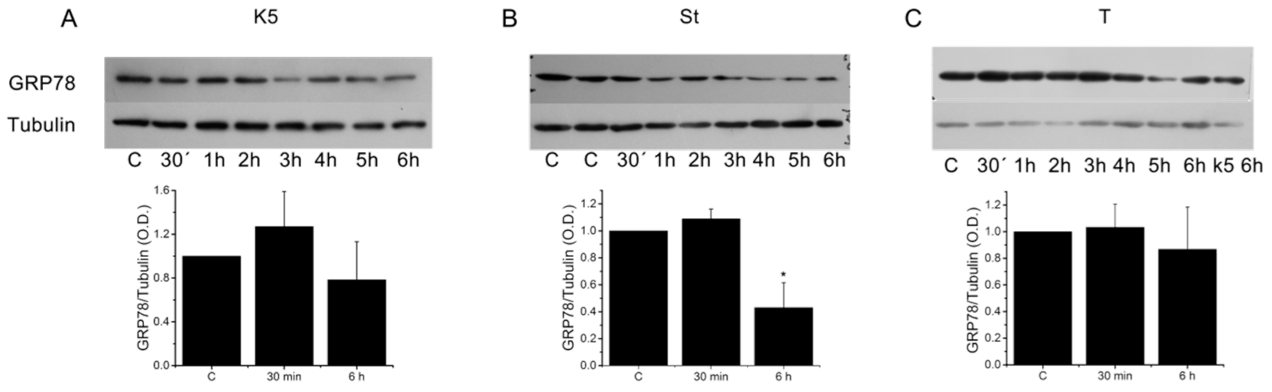

Figure S10. K5, staurosporine, or thapsigargin did not increase the expression levels of chaperone GRP78 (BiP). Control (C). CGNs in K25 were exposed to staurosporine (St, 0.5 μM) or thapsigargin (2 μM, T) or switched to K5 for 30 min up to 6 h to determine changes in the expression levels of GRP78. Images display representative blots of GRP78 (~75 kDa) and tubulin (~52 kDa), the latter used as a loading control. The ratios of GRP75/Tubulin show no increase in response to (A) K5, (B) staurosporine, and (C) thapsigargin for 30 min up to 6 h. Note that the GRP78 level did not change for the rest of the time indicated. Unexpectedly, staurosporine decreased the expression of GRP78 at 6 h in the absence of any reduction in the level of tubulin. These data suggest that the expression levels of GRP78 were not increased to cope with the induction of ER stress by these three conditions, discarding the ER stress as the main drive for CGN demise under the evaluated conditions. Data are mean ± SEM. (A) (Kruskal-Wallis, n=4). (B) (* $p < 0.05$, Kruskal-Wallis, n=6). (C) (Kruskal-Wallis, n=4).
